# Strength and hypertrophy responses to constant and decreasing rest intervals in trained men using creatine supplementation

**DOI:** 10.1186/1550-2783-8-17

**Published:** 2011-10-27

**Authors:** Tácito P Souza-Junior, Jeffrey M Willardson, Richard Bloomer, Richard D Leite, Steven J Fleck, Paulo R Oliveira, Roberto Simão

**Affiliations:** 1 Department of Physical Education. Federal University of Parana, Curitiba, Paraná, Brazil; 2 Faculty of Physical Education. State University of Campinas. Campinas, São Paulo, Brazil; 3 Kinesiology and Sports Studies Department, Eastern Illinois University, Charleston, Illinois, USA; 4 Cardiorespiratory/Metabolic Laboratory, University of Memphis, Memphis, TN, USA; 5 Physical Education Post-Graduation Program, Federal University of Rio de Janeiro. Rio de Janeiro, Brazil; 6 Sport Science Department. Colorado College. Colorado Springs, Colorado, USA

## Abstract

**Background:**

The purpose of the current study was to compare strength and hypertrophy responses to resistance training programs that instituted constant rest intervals (CI) and decreasing rest intervals (DI) between sets over the course of eight weeks by trained men who supplemented with creatine monohydrate (CR).

**Methods:**

Twenty-two recreationally trained men were randomly assigned to a CI group (n = 11; 22.3 ± 1 years; 77.7 ± 5.4 kg; 180 ± 2.2 cm) or a DI group (n = 11; 22 ± 2.5 years; 75.8 ± 4.9 kg; 178.8 ± 3.4 cm). Subjects in both groups supplemented with CR; the only difference between groups was the rest interval instituted between sets; the CI group used 2 minutes rest intervals between sets and exercises for the entire 8-weeks of training, while the DI group started with a 2 minute rest interval the first two weeks; after which the rest interval between sets was decreased 15 seconds per week (i.e. 2 minutes decreasing to 30 seconds between sets). Pre- and post-intervention maximal strength for the free weight back squat and bench press exercises and isokinetic peak torque were assessed for the knee extensors and flexors. Additionally, muscle cross-sectional area (CSA) of the right thigh and upper arm was measured using magnetic resonance imaging.

**Results:**

Both groups demonstrated significant increases in back squat and bench press maximal strength, knee extensor and flexor isokinetic peak torque, and upper arm and right thigh CSA from pre- to post-training (p ≤ 0.0001); however, there were no significant differences between groups for any of these variables. The total volume for the bench press and back squat were significantly greater for CI group versus the DI group.

**Conclusions:**

We report that the combination of CR supplementation and resistance training can increase muscular strength, isokinetic peak torque, and muscle CSA, irrespective of the rest interval length between sets. Because the volume of training was greater for the CI group versus the DI group, yet strength gains were similar, the creatine supplementation appeared to bolster adaptations for the DI group, even in the presence of significantly less volume. However, further research is needed with the inclusion of a control group not receiving supplementation combined and resistance training with decreasing rest intervals to further elucidate such hypotheses.

## Background

The combination of creatine monohydrate supplementation (CR) and resistance training has been shown to synergistically accentuate muscle fiber hypertrophy [1,2] and muscle cross-sectional area (CSA) [1]. Several studies demonstrated that CR supplementation was effective for increasing lean muscle mass, strength, muscular power, and hydration status [3-7]. Kilduff et al. [8] demonstrated that four weeks of CR supplementation in conjunction with resistance training increased maximal strength more than resistance training alone. Jonhson et al. [9] examined the influence of a loading phase of CR (20 g/day for 6 days) on bilateral leg extension repetition performance (concentric and eccentric muscle actions) until voluntary exhaustion in 18 men and women. The results indicated an approximate increase of 25% and 15% from baseline for the dominant leg in men and women, respectively. From a longitudinal standpoint, Huso et al. [10] demonstrated that 12 weeks of CR supplementation combined with resistance training increased body mass and muscle mass more than resistance training alone.

It has been suggested that CR supplementation can act through a number of distinct mechanisms. First, if phosphocreatine (PCR) concentrations are increased in skeletal muscle, PCR can then aid in the rapid rephosphorylation of adenosine diphosphate (ADP) back to adenosine triphosphate (ATP) by the CR kinase reaction during high-intensity, very short duration activities. This is especially true if the bouts of intense activity are repeated with short rest intervals in-between [11-13]. Examples of activities that derive a benefit include sprints, jumping events and weight lifting [14]. Secondly, CR supplementation can enhance the capacity for high-energy phosphate diffusion between the mitochondria and myosin heads thus, better enabling the heads to engage in cross bridge cycling and tension maintenance [11]. Thirdly, CR can act to buffer pH changes brought about by an increasing acidosis by utilizing the hydrogen ions during the CR kinase reaction and the rephosphorylation of ADP to ATP and improve cellular homeostasis. Fourthly, declining levels of PCR in the cell due to the increased need to rephosphorylate ADP can stimulate phosphfructokinase, the rate-limiting enzyme for glycolysis, thus increasing the rate of glycolysis in order to increase the rapid production of ATP [11].

The rest interval between sets is a key resistance training prescriptive variable and supplementation with CR might allow for less rest between sets, due to an enhanced capacity to restore cellular ATP concentrations between sets of fatiguing muscle actions.

Therefore, due to an enhanced recovery capacity; it is possible that CR supplementation may attenuate the decrease in performance (e.g. repetitions per set) that is often associated with shorter rest intervals between sets of resistance training. The ability to accomplish a given volume of training with less rest between sets should allow for more efficient resistance training sessions when time is limited. However, to our knowledge, no studies have examined changes in maximal strength and hypertrophy consequent resistance training programs that involve different inter-rest interval lengths in conjunction with CR supplementation [15]. Therefore, the purpose of the current study was to compare maximal strength and hypertrophy responses to resistance training programs using constant rest intervals (CI) (2-min) and decreasing rest intervals (DI) (2-min decreasing to 30-sec) between sets, during eight weeks of resistance training performed by trained men when supplementing with CR.

## Methods

### Subjects

Twenty-two recreationally trained men were randomly assigned to a constant rest interval group (CI; n = 11; 22.3 ± 1 years; 77.7 ± 5.4 kg; 180 ± 2.2 cm; 1.2 ± 0.22 bench press 1-RM/body mass; 1.42 ± 0.38 squat 1-RM/body mass) or a decreasing rest interval group (DI; n = 11; 22 ± 2.5 years; 75.8 ± 4.9 kg; 178.8 ± 3.4 cm 1.22 ± 0.26 bench press 1-RM/body mass; 1.45 ± 0.40 squat 1-RM/body mass). The inclusion criteria for participation were: a) minimum of one year resistance training experience at a frequency of four sessions per week; b) no medical conditions that could be aggravated by the training program; and c) not using any substances that may allow for a performance advantage (i.e. anabolic-androgenic steroids, other ergogenic aids). The experimental procedures were approved by the Ethics Committee of the State University of Campinas (Unicamp) and informed consent was obtained from all subjects. Additionally, subjects were asked not to perform any other structured exercise program throughout the duration of the study.

### Procedures

Pre and post testing of dependent measures was conducted over two weeks. The 1-RM tests were performed on two non-consecutive days to determine test-retest reliability. No exercise was allowed during the time between tests. The heaviest resistance lifted for the free weight back squat and bench press was considered the pre- and post-training 1-RM. These two exercises were used for strength assessment because they were common exercises performed by the subjects prior to participation in the study and the study training program utilized these two exercises. The 1-RM testing protocol has been described previously [16]. Briefly, a 1-RM was determined in fewer than five attempts with a rest interval of 5-minutes between attempts. The bench press 1-RM was determined first and then a rest interval no shorter than 10-minutes was allowed before beginning the squat 1-RM assessment.

Seventy-two hours later, muscle CSA was measured using magnetic resonance imaging. Immediately following the assessment of CSA, isokinetic peak torque was determined for the knee extensors and flexors. The test-retest reliability of the isokinetic tests was evaluated by retesting each subject six hours after the initial isokinetic test both pre- and post-training.

Knee extensor and flexor isokinetic peak torque assessments were conducted using an isokinetic dynamometer (Cybex 6000 model, Division of Lumex, Inc. Ronkonkoma, NY, USA). Subjects were positioned and stabilized in accordance with the manufacture's recommendations [17]. Before determination of the isokinetic peak torques, subjects performed a warm-up of 2 muscle actions at 60°·s^-1 ^at approximately 50% of maximum effort. After the warm-up and a rest period of 2 minutes, subjects performed a knee extensor and flexor concentric/concentric protocol of 5 maximal repetitions at the angular velocity of 60°·s^-1^. The same testing protocol was used for both the right and left legs to determine peak torque independent of the knee angle. Using the Cybex software, the greatest value was obtained during either test during both pre- and post-training and was subsequently used for the statistical analysis.

**Figure 1:**
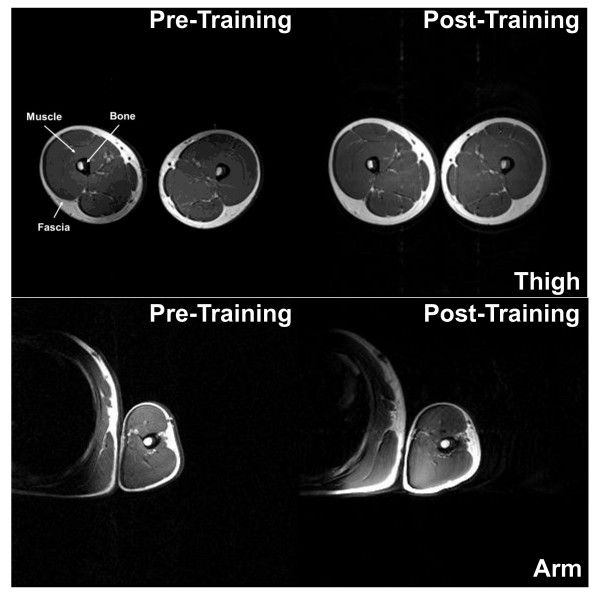
**Magnetic resonance images of the right thigh and upper arm for a single subject pre- and post-training**. Thigh and arm scan were obtained using axial T1-weighted spin-echo images with repetition time of 750 ms, echo time of 20 ms, 230 × 290 matrix resolution and number of excitations of two. Thigh images were obtained perpendicular to the femur starting at the proximal femoral epiphysis (tangential to its proximal end) and proceeding distally toward the knee joint. The slice thickness was 8 mm with no gap (forty slices) with a 45 × 45 cm field of view (FOV). Upper arm images were obtained perpendicular to the humerus starting at the proximal humeral epiphysis (tangential to its proximal end) proceeding distally toward the elbow joint. The slice thickness was 6 mm with a 1.2 mm interslice gap (forty slices) with a FOV of 40 × 32 or 40 × 40 cm depending on the arm's size.

Magnetic resonance imaging (MRI) of the right thigh and upper arm was performed using a standard body coil and a 2.0 Tesla Scanner (Elscint Prestige, Haifa, Israel) to determine muscle CSA [15] (Figure 1). The MRI equipment was calibrated prior to CSA determination of the first subject on each testing day using the manufacture's procedures. The right thigh and upper arm were scanned with subjects in a supine position. During the thigh scan the legs were relaxed and straight, feet parallel to each other and legs immobilized with pads and straps around both feet. For the upper arm scan, the arm was placed as close as possible to the magnetic iso-center aligned at the subject's side with the palm up and taped in position to the scanner bed surface.

Both the thigh and arm scan were obtained using axial T1-weighted spin-echo images with repetition time of 750 ms, echo time of 20 ms, 230 × 290 matrix resolution and number of excitations of two. Thigh images were obtained perpendicular to the femur starting at the proximal femoral epiphysis (tangential to its proximal end) and proceeding distally toward the knee joint. The slice thickness was 8 mm with no gap (forty slices) with a 45 × 45 cm field of view (FOV). Upper arm images were obtained perpendicular to the humerus starting at the proximal humeral epiphysis (tangential to its proximal end) proceeding distally toward the elbow joint. The slice thickness was 6 mm with a 1.2 mm interslice gap (forty slices) with a FOV of 40 × 32 or 40 × 40 cm depending on the arm's size. Scan time for both scans was 3 minutes and 18 seconds. The MRI images from each site were saved in a DICOM format on an optical disc and sent to a central imaging facility for analysis.

The muscle CSA of the thigh and arm was determined by manually tracing the margins of the muscles (all muscle compartments were included) and the external margin of the bone (periosteal border). The muscle CSA was obtained by subtracting the total bone area from total muscle area at pre- and post-training. Analyses were performed by the same investigator using public domain software - Image J 1.33u (National Institutes of Health, USA).

CSA of two slices per site were determined with the mean of the two slices used for statistical analyses. The slices were selected from the mid-point of the thigh and the mid-point of the arm (just distal to the deltoid insertion). To ensure that the slices analyzed pre- and post-training were taken from the same section of the thigh, the slice tangentially to the femoral head was used as an anatomical marker (first slice) and then numbered slice-by-slice distally. Two images mid-thigh were selected from each subject and their numbers recorded and used to locate the same slice during post-testing. The ninth and tenth axial slices of the thigh were selected for most subjects. The same procedure was used for the arm with the slice tangentially to the humeral head used as an anatomical marker (first slice). The twelfth and thirteenth axial slices of the arm were selected for most subjects. In two subjects, for which the number of slices between the first slice and the pre-training selected slices didn't match (different anatomical position) during pre- and post-testing, images from the pre-training were compared to the post-training scans until an identical anatomical match was found.

### Training Program

Subjects assigned to both the CI and DI groups performed the same exercises, number of sets and exercises, and repetitions per set during 8-week monitored training period. The CI group trained with 2-minute rest intervals between sets all 8-weeks, 6 days per week using 4 sets of 8-10 RM for each exercise. The exercises and training days included the following: Monday and Thursday (free-weight bench press, free-weight incline bench press, machine wide grip front lat pull down and machine seated row), Tuesday and Friday (free-weight front military press, dumbbell shoulder lateral raise, biceps barbell curl, alternating biceps curl with dumbbells, triceps extension on a pulley machine with a v-shaped handle and lying triceps extension with a barbell), and Wednesday and Saturday (free-weight back squat, leg extension machine, leg curl machine and abdominal crunch).

The training program for the DI group consisted of the same exercises, days of training per week, and number of sets and repetitions. The only difference in training programs was the rest interval. The DI group started with a 2 minute rest interval the first two weeks, after which the rest interval between sets was decreased 15 seconds per week (i.e. first and second weeks - 2 minutes; third week - 105 seconds; fourth week - 90 seconds; fifth week - 75 second; sixth week - 60 seconds; seventh week - 45 second; and eighth week - 30 seconds). The gradual reduction in rest interval length was to allow the subjects gradual adjustment to better tolerate the shorter rest intervals. Prior to each training session, subjects in both groups performed a warm-up consisting of two sets of 20 repetitions with 50% of the load used for the first exercise of the session.

In both groups, each training session was supervised by an experienced strength and conditioning professional and subjects were verbally encouraged to perform all sets to voluntary exhaustion. The training load was adjusted as necessary to stay within the 8-10 RM range. There was no attempt to control movement velocity. Adherence to the program was 100% for subjects in all groups. The mass of all weight plates and bars used for training was determined with a precision scale (Filizola Balanças Industriais S.A., São Paulo, Brazil). The machine exercises were performed using strength training machines (Life Fitness Inc., Franklin Park, IL, U.S.A.). The weekly volume achieved for the free weight bench press and back squat was calculated as the sum of the load lifted, multiplied by the total repetitions for the two workouts performed during each week for both exercises.

### CR Supplementation

The study was conducted in a double-blind manner in which subjects ingested capsules orally. In the first week of supplementation, subjects in both groups began the loading phase (7 days) consuming 20 g of CR plus 20 g maltodextrin per day divided into four equal dosages of 10 g (5 g of CR + 5 g of maltodextrin). After the loading phase and until the end of the study (35 days), the supplement was consumed in a single dose immediately following the training session (5 g of CR + 5 g of maltodextrin). The protocol of supplementation was adapted from Volek et al. [2]. The supplements (CR and maltodextrin) used were provided by ATP Brasil Com. LTDA (Campinas, São Paulo, Brazil). The subjects' diets were not standardized; however, all subjects were instructed to maintain their normal dietary habits during the course of the study. Compliance to the supplementation protocol was monitored by verbal confirmation and all subjects recorded supplementation time in accordance with the investigators' instructions. At the time of the pre-test, all subjects submitted a dietary recall for two days during the week and one day on the weekend; after that, subjects were instructed to maintain the same dietary consumption during experimental period.

### Statistical Analysis

Intra-class correlation coefficients (ICC) were used to determine the test-retest reliability for the 1-RM, isokinetic peak torque, and muscle CSAs data. Student's t-tests were also used to assess differences between test/retest scores for all dependent measures pre and post intervention. The statistical analysis was initially done using the Shapiro-Wilk normality test and the homocedasticity test (Bartlett criterion). Two way ANOVAs (time [baseline vs. 8 weeks training] × group [CI vs. DI]) with repeated measures, followed by Tukey's post hoc tests (in the case of significant Main Effects), were used to assess significant differences (p < 0.05) between groups for dependent variables: 1-RMs, muscle CSAs, isokinetic peak torques, and weekly training volume for the free-weight bench press and back squat. The scale proposed by Cohen [18] was used for classification of the effect size magnitude (the difference between pretest and post-test scores divided by the pre-test standard deviation) of 1-RMs, muscle CSAs, isokinetic peak torques. Statistica version 7.0 (Statsoft, Inc., Tulsa, OK) statistical software was used for all statistical analyses.

## Results

Pre- and post-training, the 1-RM bench press (r = 0.96, r = 0.96) and back squat (r = 0.90, r = 0.92) tests showed high intra-class correlation coefficients, respectively and the paired t-tests indicated no significant differences. The test-retest reliability of the isokinetic pre- and post-training peak torque assessment of the knee extensor (r = 0.96, r = 0.96) and flexor (r = 0.96, r = 0.96) tests showed high intra-class correlation coefficients, respectively and the paired t-tests indicated no significant differences. The reproducibility of CSA measurements was evaluated by analyzing each subject's arm and thigh image. The test-retest reliability of the CSA for both the thigh pre and post-training (r = 0.97; r = 0.97) and arm (r = 0.99; r = 0.99) showed high intra-class correlation coefficients, respectively and the paired t-tests indicated no significant differences.

**Figure 2:**
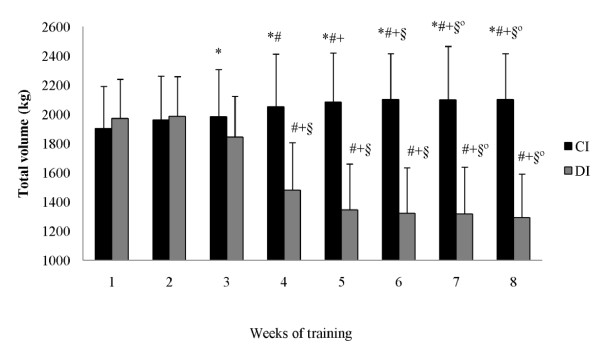
**Bench press total training volume at each week of training (mean ± SD)**. CI = constant rest interval group; DI = decreasing rest interval group. * = significant difference between the groups. ^# ^= significant difference to 1^st ^week. ^+ ^= significant difference to 2^nd ^week. ^§ ^= significant difference to 3^rd ^week. ^@ ^= significant difference to 4^th ^week.

**Figure 3:**
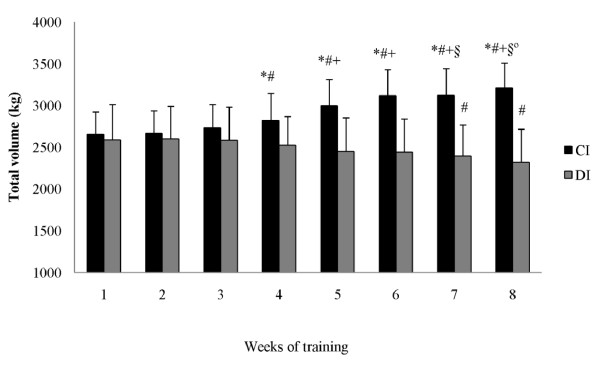
**Squat total training volume at each week of training (mean ± SD)**. CI = constant rest interval group; DI = decreasing rest interval group. * = significant difference between the groups. ^# ^= significant difference to 1^st ^week. ^+ ^= significant difference to 2^nd ^week. ^§ ^= significant difference to 3^rd ^week. ^@ ^= significant difference to 4^th ^week.

There were no significant differences between groups prior to the intervention in the anthropometric, strength, or muscle CSA measures. Neither group demonstrated a significant change in total body mass from pre- to post-training. The total training volume (load × repetitions) for the bench press during the 8-week training program was significantly greater (22.9%) for the CI group compared to the DI group (Figure 2). Similarly, the total training volume for the back squat was significantly greater (14.6%) for the CI group compared to the DI group (Figure 3).

Both groups showed significant increases in bench press and squat 1-RM (Table 1), knee extensor and flexor isokinetic peak torque pre- to post-training (Table 2) and muscle CSA (Table 3); however, there were no significant differences between groups for any of these variables. The ES data demonstrated similar magnitudes for bench press and squat 1-RM (Table 1) and knee extensor and flexor isokinetic peak torque pre- to post-training (Table 2). However, the ES for upper arm and right thigh CSAs presented large magnitudes for the DI (Table 3).

**Table 1:** One repetition maximum loads (mean ± SD) and Effect Sizes for bench press and squat exercises.

	Bench press			Squat		
	
	Pre (kg)	Post (kg)	ES	Pre (kg)	Post (kg)	ES
CI	102 ± 10	130 ± 10*	2.80 (large)	115 ± 20	155 ± 20*	2.00 (large)
DI	100 ± 12	125 ± 12*	2.08 (large)	120 ± 22	160 ± 15*	1.81 (large)

ES = Effect Size; CI = constant rest interval group; DI = decreasing rest interval group. * Statistically significant difference (p ≤ 0.0001) between pre-training and post-training.

**Table 2:** Isokinetic knee flexor and extensor peak torque (N.m) values (mean ± SD) and Effect Sizes.

	Knee flexor			Knee extensor		
	
	Pre (N**^.^**m)	Post (N**^.^**m)	ES	Pre (N**^.^**m)	Post (N**^.^**m)	ES
CI						
*Right*	128.8 ± 22	144 ± 30*	0.69 (moderate)	248.2 ± 22	268.4 ± 10*	0.92 (moderate)
*Left*	130.5 ± 20	145.4 ± 28*	0.75 (moderate)	246.4 ± 28	256.5 ± 12*	0.36 (small)
DI						
*Right*	128.5 ± 18	138.0 ± 19*	0.53 (small)	244.0 ± 20	258.0 ± 25*	0.70 (moderate)
*Left*	126.2 ± 22	138.4 ± 16*	0.56 (small)	236.0 ± 14	245.4 ± 24*	0.67 (moderate)

ES = Effect Size; CI = constant rest interval group; DI = decreasing rest interval group. * statistically significant difference (p ≤ 0.0001) between pre-training and post-training.

**Table 3:** Muscle cross-sectional area of the upper arm (CSAA) and right thigh (CSAT) values (mean ± SD) and Effect Sizes.

	CSAA (cm**^2^**)			CSAT (cm**^2^**)		
	
	Pre	Post	ES	Pre	Post	ES
CI	65.2 ± 8.0	74.2 ± 6.5 *	1.11 (moderate)	170.4 ± 15.9	202.4 ± 22.1*	2.02 (large)
DI	63.5 ± 5.2	76.7 ± 4.2 *	2.53 (large)	166.4 ± 14.2	212.2 ± 20.2 *	3.23 (large)

ES = Effect Size; CI = constant rest interval; DI = decreasing rest interval. *statistically significant difference (p ≤ 0.0001) between pre-training and post-training. 0.2, 0.6, and 1.2 for small, moderate, and large

## Discussion

The main findings of the present investigation were: 1) the combination of CR supplementation and structured resistance training increased muscular strength, isokinetic peak torque, and muscle CSA, irrespective of the rest interval length between sets, 2) progressively decreasing the rest interval length between sets, although not negatively impacting muscular strength and CSA adaptations to resistance training, significantly impaired exercise acute repetition performance within a given workout (more for upper body exercise than for lower body exercise), and 3) it did not appear as though CR supplementation attenuated the decrease in acute repetition performance with progressively shorter rest intervals between sets. However, based on this final statement, our failure to include a true control group not receiving CR supplementation but undergoing a progressive decrease in rest interval length does not allow us to make such a statement with absolute confidence, regarding the ability of CR to off-set any additional decrease in training volume that may have been apparent. This is indeed a limitation of the present work and should be a focus of future research.

A previous study from our research group [15] compared the effect of 8-weeks of resistance training using CI and DI between sets and exercises on strength and hypertrophy. Recreationally resistance training subjects were randomly assigned to either a CI or DI training group. The results indicated no significant differences between the CI and DI training protocols for CSA, 1RM and isokinetic peak torque. Similar to the current study, these results [15] indicated that a training protocol with DI was as effective as a CI protocol over short training periods (8-weeks) for increasing maximal strength and muscle CSA.

Muscle mass is important for health and survival through the lifespan [7]. Resistance training has been recognized as an essential component of a comprehensive fitness program for individuals with diverse fitness goals [19]. Manipulation of training variables (e.g. load, volume, rest interval between sets) is dependent on the specific training goals of the individual and the nature of the physical activities performed during daily life [20,21]. The length of rest interval must be sufficient to recover energy sources (e.g., adenosine triphosphate [ATP] and PCR), buffer and clear fatigue producing substances (e.g., H^+ ^ions), and restore force production [22].

Certain ergogenic substances have been shown to augment resistance training adaptations beyond that which may occur through resistance training alone. With regard to the function of the Phosphagen energy system, the ergogenic value of CR supplementation has been examined extensively with significant benefits reported in strength/power, sprint performance, and/or work performed during multiple sets of maximal effort muscle contractions [1,2,23-25]. The improvement in exercise capacity has been attributed to increased total creatine (TCR) and PCR content, thus resulting in greater resynthesis of PCR, improved metabolic efficiency and/or an enhanced quality of training; thus promoting greater neuromuscular adaptations.

The increased muscle strength and improved weightlifting performance following CR ingestion plus resistance training could result from several mechanisms, including greater gains in lean body mass [2] and an increase in the intensity of individual workouts, resulting from a better ability to meet energy demands during exercise [26]. We contend that the beneficial effects of CR supplementation on muscle strength and weightlifting performance during resistance training are largely the result of the CR-loaded subjects ability to train at a higher workload than placebo-supplemented subjects, as suggested previously [27,28]. However, while this may be the case when maintaining rest interval length, our present data indicate that when rest interval length is decreased significantly, the total training load is decreased despite CR supplementation.

Although we did not include a true control group that did not receive CR supplementation but underwent training using a progressively decreasing rest interval; it is plausible that CR may attenuate the decrease in training volume when subjects are exposed to such a condition. Regardless, and perhaps of most importance to athletes who use CR for purposes of increasing strength and muscle mass, the volume of training was greater for the CI group versus the DI group but strength gains were similar between groups. Thus, the creatine supplementation appeared to bolster strength gains particularly for the DI group, even in the presence of significantly less volume. However, future work is needed to investigate the relationship between CR supplementation versus no supplementation on volume parameters and strength and muscle mass increases during long term studies.

In long-term studies, subjects taking CR typically gain about twice as much body mass and/or fat free mass (i.e., an extra 2 to 4 pounds of muscle mass during 4 to 12 weeks of training) versus subjects taking a placebo [29,30]. The gains in muscle mass appear to be a result of an improved ability to perform high-intensity exercise via increased PCR availability and enhanced ATP synthesis, thereby enabling an athlete to train harder to promote greater muscular hypertrophy via increased myosin heavy chain expression; possibly due to an increase in myogenic regulatory factors myogenin and MRF-4 [31-33]. In the present study, we clearly noted a reduction in training volume for the DI group.

We speculate that because the loads for the current study were in the 8-10 RM range, perhaps anaerobic glycolysis was being emphasized to a greater extent for ATP production. As the rest intervals were progressively shorter in the DI group, there would have been limited time to resynthesize PCr, and greater reliance would have been placed on rapid glycolysis to effectively meet energy demands. Therefore, creatine supplementation might be more effective in maintaining volume with higher loads and less repetitions per set (e.g. one to six repetition maximum per set). Despite this, subjects in the DI group maintained similar adaptations in muscle strength and CSA as compared to subjects in the CI group. It is possible that subjects' overall perceived effort and intensity plays a significant role in the adaptive process, as opposed to simply the absolute volume load. That is, all subjects adapted to a similar degree, yet those in the DI group demonstrated significant reductions in volume load versus the CI group (see Tables 1 and 2).

According to the Position Statement of International Society of Sports Nutrition, CR monohydrate (and not other forms of CR) is the most effective ergogenic nutritional supplement currently available to athletes in terms of increasing high-intensity exercise capacity and lean body mass during training [4]. To date, several hundred peer-reviewed research studies have been conducted to evaluate the efficacy of CR supplementation in improving exercise performance. Nearly 70% of these studies have reported a significant improvement in exercise capacity, while the others have generally reported non-significant gains in performance [34].

Arciero et al. [35] compared 1-RM strength gains after 4 weeks of CR supplementation with or without resistance training. Bench press and leg press 1-RM were increased 8 and 16%, respectively, in the CR alone group and 18 and 42%, respectively, in the training group. This study suggests that approximately 40% of the increase in strength over the 4-week training and CR supplementation period is due to the acute effects of CR on force production, with the remaining 60% due to some other mechanism, presumably an ability to train with higher workloads. Syrotuik et al. [36] reported that when training volume is equal, subjects ingesting CR or placebo experienced similar increases in muscle strength and weightlifting performance following an 8-week resistance training program. Thus, it is probable that subjects who ingest CR during resistance training do more work than those who do not [32,33]. Again, this assumes that rest interval length remains constant, unlike the present design.

Larson-Meyer et al. [27] conducted a double-blind, placebo-controlled study, which involved 14 division I female soccer players during their 13-week off-season resistance training program. Seven of the women were CR loaded with approximately 7.5 g twice daily for 5 days, and then maintained their CR intake at 5 g/day for the remainder of the study. Following a repeated measures analyses to establish trial by group interactions, it was determined that bench-press and squat 1-RM strength improved more for the CR group compared with the placebo group. There was, however, no difference between the two groups concerning overall gains in lean tissue as determined by dual energy x-ray absorptiometry (DXA).

To our knowledge, the current study was the first to compare the chronic effects of CR supplementation in a training program using decreasing rest intervals between sets and exercises to a program using constant rest intervals. In strength-type regimens, the recommended rest interval of 2-5 minutes between sets has been shown to allow for consistent repetitions, without large reductions in the load [37-40]. Conversely, in hypertrophy-type regimens, the recommended rest interval of 30-90 seconds is not sufficient to sustain the load and/or repetitions over consecutive sets [41,42]. Our data clearly indicate that, despite CR supplementation, reduction of rest interval length below 105 seconds (week 4; 90 seconds) significantly impairs exercise performance (in particular as related to bench press performance).

The need for longer rest intervals when emphasizing strength are supported by Pincivero et al. [43] for isokinetic training with either 40 seconds or 160 seconds rest between sets. One leg of each subject was assigned to a four week, three days per week isokinetic protocol that involved concentric knee extension and flexion muscle actions performed at 90°·s^-1^. The 160 second rest group demonstrated significantly greater increases in quadriceps and hamstring peak torque (60°·s^-1^), average power (60°·s^-1^), and total work (30 repetitions at 180°·s^-1^).

In the current study, despite a decrease in training volume load in the DI group, both groups showed significant increases pre- to post-training in knee extensor and flexor isokinetic peak torque. No significant difference between the DI and CI groups in peak torque at an angular velocity of 60°·s^-1 ^was shown indicating isokinetic peak torque is equally increased with both CI or DI training groups.

Robinson et al. [37] demonstrated findings that were consistent with Pincivero et al. [43] for free weight training. In this study, the effects of three different intervals (3 minutes, 90 seconds and 30 seconds) were compared on maximal back squat strength. Thirty-three moderately trained college age men performed a free weight training program four days per week for five weeks. The group that rested 3 minutes between sets demonstrated significantly greater increases in maximal back squat strength versus the 90 second and 30 second rest groups.

Conversely, Willardson and Burkett [44] compared back squat strength gains and volume components in 15 recreationally trained men that were divided into a 2 minute rest group and a 4 minute rest group. Each group performed the same training program, with the only difference being the length of the rest interval between sets. Subjects performed two squat workouts per week. The squat workouts varied in the load, number of sets, and repetitions performed per set in a nonlinear periodized manner. Differences in strength gains and volume components (the load utilized per set, the repetitions performed per set, the intensity per set, and the volume performed per workout) were compared between groups. The key finding was that during the entire training period; the 4 minute group demonstrated significantly greater total volumes during the higher intensity workouts. However, the groups were not significantly different in back squat strength gains. These findings suggest that there was a threshold in terms of the volume necessary to gain a certain amount of strength, similar to the current study in which the DI group made similar strength gains as the CI group.

Creatine supplementation has multiple metabolic effects and may possibly influence the hormonal response to exercise and subsequent hypertrophy [7]. If so, this may help to explain our findings of improved muscle strength and CSA despite a reduction in training volume load for the DI group. Ahtiainen et al. [45] indicated that hormonal responses and hypertrophic adaptations did not vary with 2 or 5 minute rest intervals in 13 recreationally trained men (with an experience of 6.6 ± 2.8 years of continuous strength training). This experiment involved a cross-over design so that two groups trained 3 months with each rest condition. The maximal strength of the leg extensors and quadriceps CSA was assessed before and after completion of each condition. Other variables that were assessed included: electromyographic activity of leg extensor muscles, concentrations of total testosterone, free testosterone, cortisol, growth hormone, and blood lactate. The results demonstrated that for both conditions, acute responses and chronic adaptations were similar in terms of the hormonal concentrations, strength development, and increases in quadriceps CSA. A key finding by Ahtiainen et al. [45] was that the 5 minute rest interval allowed for the maintenance of a higher training intensity (approximately 15% higher); however, the volume of training was equalized so that the 2 minute condition required more sets at a lower intensity, while the 5 minute condition required less sets at a higher intensity. Thus, the strength and hormonal responses appeared to be somewhat independent of training intensity as long as an equal volume was performed.

Buresh et al. [46] also compared the chronic effects of different inter-set rest intervals after 10 weeks of strength training. Twelve untrained males were assigned in strength training programs using either 1- or 2.5-minute rest between sets, with a load that elicited failure only on the third set of each exercise. Measures of body composition, hormone response, thigh and arm indirectly CSA, and 5 RM loads on squat and bench press were assessed before and after 10 weeks program. The results showed that 10 weeks of both strength training programs resulted in similar significant increases in 5 RM squat and bench press strength, thigh and arm CSA, and lean mass. However, 1-minute of rest between sets elicited a greater hormonal response versus 2.5-minutes of rest between sets during the first training weeks, but these differences disappeared after 10 weeks of training. These results suggested that acute hormonal responses may not necessarily be predictive of hypertrophic gains after 10 weeks training program performed by untrained healthy males [46]. Considering all available evidence, it appears that multiple factors are involved in strength and hypertrophy development, including but likely not limited to perceived subject effort, training volume, training intensity, metabolic factors associated with recovery, and acute and long-term hormonal responses.

## Conclusions

In the present study, it is important to highlight that ES for upper arm and right thigh CSAs presented large magnitudes in DI. These data support that decreasing interval seems to be more efficient than constant interval to produces hypertrophic responses. However, more work is needed in this area to tease out the specific contributions of each component. In conclusion, we report that the combination of CR supplementation and resistance training can increase muscular strength, isokinetic peak torque, and muscle CSA, regardless of rest interval length. When decreasing rest interval length, although not negatively impacting muscular strength, a significant impairment in exercise performance is observed, despite CR supplementation. Future studies, inclusive of a true control group not receiving CR supplementation but undergoing training using decreased rest interval length, are needed to determine whether or not CR supplementation can attenuate the decrease in training volume observed when rest interval length is decreased.

## Competing interests

All researchers involved impartially collected, analyzed, and interpreted the data from this study and have no financial interests concerning the outcome of this investigation. The results from this study do not represent support by the authors and their institutions concerning the supplement investigated

## Authors' contributions

TPSJ conceived of and designed this study, contributed to the acquisition, analysis and interpretation of data, led the drafting and revising of the manuscript. JMW involved in drafting the manuscript and revising of the manuscript. SJF conceived of the study, and participated in its design and helped to draft the manuscript. PRO conceived of and designed this study, contributed to the acquisition, analysis and interpretation of data. RDL Assisted data interpretation and manuscript preparation. RS Assisted the design of the study, data interpretation and manuscript preparation.

RB involved in drafting the manuscript and revising of the manuscript. All authors have read and approved the final manuscript.
